# DigSee: disease gene search engine with evidence sentences (version cancer)

**DOI:** 10.1093/nar/gkt531

**Published:** 2013-06-11

**Authors:** Jeongkyun Kim, Seongeun So, Hee-Jin Lee, Jong C. Park, Jung-jae Kim, Hyunju Lee

**Affiliations:** ^1^School of Information and Communications, Gwangju Institute of Science and Technology, 123 Cheomdangwagi-ro, Buk-go, Gwangju 500-712, Republic of Korea, ^2^Department of Computer Science, KAIST, 291 Daehak-ro, Yuseong-gu, Daejeon 305-701, Republic of Korea and ^3^School of Computer Engineering, Nanyang Technological University, Blk N4-02B-44, Nanyang Avenue, Singapore 639798

## Abstract

Biological events such as gene expression, regulation, phosphorylation, localization and protein catabolism play important roles in the development of diseases. Understanding the association between diseases and genes can be enhanced with the identification of involved biological events in this association. Although biological knowledge has been accumulated in several databases and can be accessed through the Web, there is no specialized Web tool yet allowing for a query into the relationship among diseases, genes and biological events. For this task, we developed DigSee to search MEDLINE abstracts for evidence sentences describing that ‘genes’ are involved in the development of ‘cancer’ through ‘biological events’. DigSee is available through http://gcancer.org/digsee.

## INTRODUCTION

As human complex diseases are caused by multiple genes with low penetrance, extensive efforts have been made to find disease-associated genes, resulting in the accumulation of millions of biomedical articles. Manually curated databases by experts that contain such gene–disease associations from those articles are important resources for disease research. GeneCards (1) is one of the largest databases of genes and lists disease-associated genes by integrating several disease databases (NCBI, ENSEMBL, etc). Human Gene Mutation Database (2) provides a list of genes, mutations of which cause diseases. Other databases are designed for specific diseases, including Dragon databases of genes associated with esophageal cancer and prostate cancer (DDEC, DDPC) (3,4). Web-based biomedical text-mining systems for general purposes such as Polysearch (5), Génie (6), FACTA (7), G2D (8) and GeneView (9) can be used to search for association between genes and diseases. For example, Polysearch (5) retrieves and analyzes PubMed results, and then integrates the search results with other databases [DrugBank (10), Human Gene Mutation Database, etc] to improve the accuracy of the association between diseases and genes.

Although those tools provide information about whether a gene is related to a disease in any way, the information is not specific enough to explain the molecular context of how the gene affects the disease. Understanding the gene–disease relation can be further enhanced by identifying in which biological events (e.g. gene expression, regulation, phosphorylation, localization and protein catabolism) the genetic effect is valid for the disease development. As such information of molecular context is abundantly reported in the literature, we propose to use fine-grained information extraction techniques to retrieve the specific information from the literature.

Although a few databases collect biological events of a single type such as methylation in cancer [e.g. MeInfoText (11), PubMeth(HL) (12)], there is no specialized text-mining tool yet allowing for a query into the explicit triple relationship among diseases, genes and biological events. Instead, the biomedical text-mining community has recently paid great attention to extracting the binary relations between genes and biological events (13). We use one of the state-of-the-art event extraction systems (14) and develop a novel text-mining method to further identify the relations of the extracted events and genes with diseases, which are explicitly expressed in sentences.

Our novel search engine, DigSee, services the sentences with those identified triple relations, on the requests from users, which require information such that ‘which genes’ are involved in the development of ‘which disease’ through ‘which biological events’. We refer to a gene as a disease-related gene (or disease gene for simplicity) if it is either directly or indirectly related to the cause of disease or contributes to increasing or decreasing the properties of disease in cell. DigSee collects and ranks sentences, called evidence sentences, which explicitly express that the disease gene changes the properties of disease cell through biological events. For example, if the molecular event of a gene increases cancer-related properties such as angiogenesis or cell proliferation, we recommend it as a cancer-related gene. The goal of our work is to identify these evidence sentences, demoting sentences that do not show relation between gene and disease.

**Figure 1.**
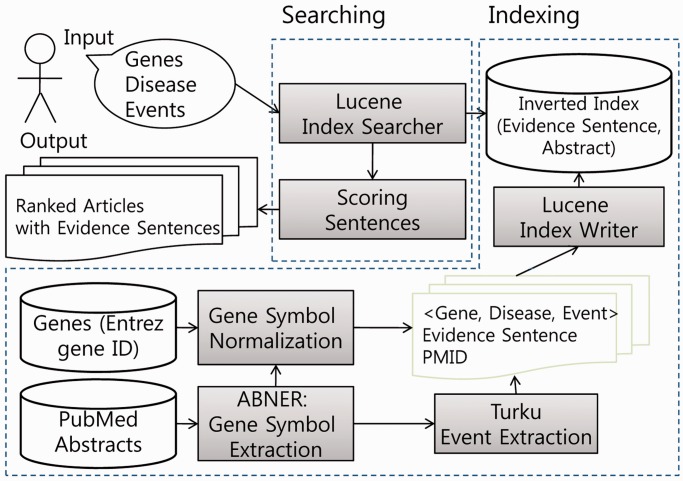
Indexing and searching processes in the DigSee system.

In the rest of the article, we explain each of the three components of DigSee, including (i) a Web-based user-query interface and a display tool to present search results, (ii) an indexing process to extract gene symbols and events from text and store them into inverted indexes and (iii) a searching process to find evidence sentences from documents for a given query and to score relevance of evidence sentences. Figure 1 depicts the workflow of the system.

## RESULTS

The current version of DigSee supports all cancer types, the worldwide leading cause of death, and the following event types as the molecular context of gene–disease association: gene expression, transcription, phosphorylation, localization, regulation, binding and protein catabolism. We have collected 1 391 019 evidence sentences from cancer-related MEDLINE abstracts, where sentences contain at least one gene name with Entrez gene ID and one event extracted by the Turku event extraction system (14) and may express the triple relation among a cancer, the gene and the event. We introduce some example queries for DigSee and their resultant evidence sentences, which may show the biological significance of the tool.

### Example queries

Example queries provided in the DigSee Web site include the followings:

- Jia *et al.* (2011) (15) identified 124 differentially expressed genes on prostate cancer using microarray experiment. Authors might be interested in how many of 124 genes were previously reported in the literature such that their expression changes were related to prostate cancer development. Show evidence sentences supporting which genes were known to be related to prostate cancer,
- Show evidence sentences for the association of TP53 gene with colon cancer through any of the seven biological event types and
- Show a list of genes whose localization and transcription are related to brain cancer.

Microarray experiments such as the one in example (i) often identify more than hundreds of candidate cancer-related genes. Finding literature evidence of these genes is the first step to validate them. For the query (i), DigSee returns evidence sentences for 35 genes of the 124 genes, which are highlighted with the expression changes of the candidate genes in prostate cancer. If we change the query to select both regulation and gene expression as events, 49 genes are searched with evidence sentences. In the example (ii), TP53 is a well-known tumor suppressor gene. This query is useful in finding in which molecular context TP53 plays a critical role in colon cancer. The query returns evidence sentences for all of seven events. An example of evidence sentences is ‘Overexpression of 15-lipoxygenase-1 induces growth arrest through phosphorylation of p53 in human colorectal cancer cells.’ from an article (16) explaining a mechanism how phosphorylation of TP53 induced by overexpression of 15-LOX-1 affects growth arrest. In the example (iii), DigSee system returns a list of 394 genes with evidence sentences in which localization and transcription events of these genes are related to brain cancer. Some of examples include that ING1 proteins are aberrantly localized to the cytoplasm in brain cancer (17), and that eukaryotic initiation factors 2alpha and 4E are frequently localized in the nucleus of meningiomas and in the same compartment of the oligodendroglial tumors, respectively (18).

### Web interface of DigSee system

DigSee’s query consists of the following elements: (i) a disease of interest, currently a human cancer (compulsory); (ii) candidate gene(s) that might be related to the disease (if no gene is given in the query, all genes will be considered as candidates) and (iii) event type(s) out of the seven types that might explain the molecular context of the gene–disease association. The three elements together represent a user request for evidence sentences that describe the role of ‘genes’ in the development of ‘disease’ through ‘biological events’. Synonyms and subtypes of cancer collected from GeneCards are also considered in the DigSee system.

**Figure 2.**
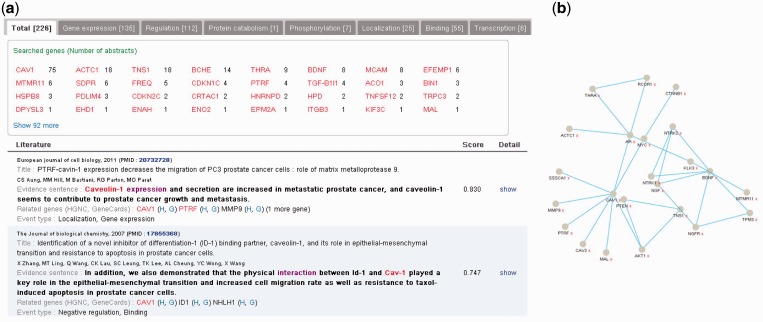
Screenshots of the Web interface of the DigSee system. (**a**) Main search results are MEDLINE abstracts with evidence sentences supporting that genes are related to a given cancer type through biological events. (**b**) A graph visualizes genes with evidence sentences. Nodes are genes and two nodes are connected if they appear in the same documents. When the number of genes is large, only a subset of genes with high evidence sentence score is shown. By increasing a threshold for the number of genes, more genes will appear. Users can expand neighbor genes by clicking a right button for a node. Clicking an edge will show a list of abstracts in which two genes appear together.

The display tool presents search results consisting of MEDLINE abstracts with evidence sentences. As shown in Figure 2, one sentence with the max score is shown along with citation information for each abstract, and abstracts are sorted according to their sentences’ scores. In each sentence, input gene names and event keywords are highlighted. By following a Detail link, users can check all evidence sentences in an abstract, including evidence sentences for other cancer-related genes as well as those for input genes. Links to gene entries in such databases as HUGO Gene Name Consortium and GeneCards are also provided. When an input query has multiple genes or multiple event types, a separate display for each gene or event type is provided. In addition, sentence-level co-occurrences among genes are visualized in a graph, which might give users insights into the disease pathway. All searched results can be downloaded to text files.

### Statistics of DigSee system

First, 3 010 235 cancer-related articles were retrieved from PubMed (as of 8 February 2013). Among them, 1 993 518 remained after excluding books or articles without abstracts. Finally, 1 099 819 abstracts from MEDLINE were indexed for ∼200 cancer names after selecting abstracts containing evidence sentences. Using our proposed method, 1 391 019 sentences were scored. The numbers of scored sentences by cancer types and by biological events are shown in a help page of the DigSee Web site.

## METHODS

### Indexing and searching

The indexing process of the DigSee system is depicted in Figure 1. It collects abstracts from PubMed using general cancer terms such as ‘cancer’, ‘tumor’, ‘neoplasm’, ‘carcinoma’ and ‘sarcoma’, and specific cancer names not including those general terms such as ‘glioblastoma’ and ‘leukemia’. Then, it constructs two inverted indexes, an abstract index and an evidence sentence index. These inverted indexes contain locations of abstracts and evidence sentences in the file system for cancer terms, gene symbols and biological events so that they would allow fast abstract/sentence searches with these queries. We consider a sentence with a gene and a biological event as a candidate evidence sentence, where a cancer keyword can be found either in the sentence or in the same abstract. The abstract index has links to candidate evidence sentences in the retrieved abstracts. The evidence sentence index contains information of gene(s), a cancer type and biological event(s) in each sentence.

A biological named entity recognition (NER) system, ABNER (19), was used to find genes and proteins in biomedical literature. ABNER achieved a recall of 65.9%, a precision of 74.5% and an F-measure of 69.9%, when evaluated against 2500 sentences from the BioCreative protein/gene corpus (20). Another NER system, BANNER (21), was also considered. We tested the two NER systems against 25 abstracts from the abstract index and found that their accuracies were similar.

The recognized gene/protein names were normalized using Moara (22), which participated in the BioCreative II human gene normalization task (23) and achieved a precision of 55%, a recall of 83.31% and an F-measure of 66.26%. Moara uses gene synonyms from UniProt (24) and the HUGO Gene Name Consortium (25) and, although not the best-performing system, has several advantages such that it can be used to normalize a gene name alone as in a DigSee query, and that it is fast and freely available. On the other hand, other tools such as GNAT (26) combine gene name extraction and gene normalization steps so that they cannot be used for the normalization of DigSee query genes. Note that Moara (22) was also used for the query gene normalization.

The Turku event extraction system (14) was used to locate biological events. It was the best-performing event extraction system in the BioNLP’09 shared task (13) and achieved an F-measure of 52.86% (precision 58.13% and recall 48.46%). It requires an NER system and a parser; we used ABNER (19) and Stanford parser (27). It extracts complex events among genes and proteins from biomedical literature. It consists of three major processing steps: trigger recognition, edge detection and semantic post-processing. First, the system finds triggers (or keywords) of biological events. Then, it predicts relation edges between named entities and event triggers, which form into a semantic graph. The trigger recognition and edge detection steps are based on multi-class support vector machine (SVM) classifiers. In the semantic post-processing step, the system refines the edges of the semantic graph using a rule-based model. The Turku event extraction system identifies nine events: protein catabolism, phosphorylation, transcription, localization, regulation, binding, negative regulation, gene expression and positive regulation. In DigSee, positive regulation and negative regulation are combined into a single type of regulation, because the event polarity does not always indicate the polarity of the causal relation between disease and gene.

The Turku event extraction system identifies incorrect events in some cases. When we manually checked the relevance of the identified events, some event keywords are constantly found irrelevant to a given event type in almost all the sentences. Therefore, we included a pre-screening step to filter out these irrelevant event keywords from the event-word index of the DigSee system. For example, words such as ‘described’, ‘derived’, ‘prescribed’ and ‘transition’ are almost always irrelevant to transcription events in our data sets. Also, the chromosomal location of a gene is removed from ‘Localization event’ because we focus on sub-cellular localization events or the presence of a protein in the cell. We provide the list of these filtered-out words for all the seven events in a Web page (named as Supplementary page) at http://gcancer.org/digsee/supple.html. Note that this list is not complete yet, and we will continue to update it.

In the searching process (Figure 1), indexed evidence sentences are searched for an input query. The evidence sentence index returns sentences that contain genes, cancer and events of the input query. When the query contains no gene, any genes with the given biological events in a given cancer type will be retrieved. Abstracts are sorted by the scores of evidence sentences. A scoring method for the relevance of evidence sentences is presented in the next section. Apache Lucene search engine library was used for building the indexing and searching processes.

### Ranking

The ranking step is to measure the relevance of the candidate evidence sentences, which means whether the recognized gene is the subject of the identified event that leads to changes of the given disease’s properties. We developed a machine learning model for the ranking, which is trained on a gold-standard data set manually constructed by the authors. We introduce the gold-standard data set and the features of the machine learning model in this subsection.

### Gold-standard data

The gold-standard data set consists of a subset of candidate evidence sentences, which are manually classified into either positive or negative evidence. A sentence is positive if it contains information that a gene is involved in cancer development through an event. Note that the cancer term can appear in the same sentence as the gene and event or in another sentence in the same abstract. A sentence is classified as negative if a gene is not involved in cancer development, or an event is not related to the gene or cancer. A sentence is also classified as negative if it contains incorrectly identified gene symbols and events due to the faults of the NER system and the event extraction system.

Sentences 1 and 2 obtained from two abstracts (28,29) are positive and negative evidence sentences, respectively. Sentence 1 contains information that the gene SOX9 is involved in development of prostate cancer through down-regulation. On the other hand, Sentence 2 describes the purpose and procedure of an experiment without explicitly mentioning the experiment results. We will use these two sentences to explain the features of our ranking method.

- **Sentence 1** Significantly, down-regulation of SOX9 by siRNA in prostate cancer cells reduced endogenous AR protein levels, and cell growth indicating that SOX9 contributes to AR regulation and decreased cellular proliferation.
- **Sentence 2** To determine the role of CD147 in the invasiveness properties of prostate cancer, we successfully down-regulated CD147 by RNA interference (RNAi) technology, in PC-3 cell line at high level of CD147 expression.

The total number of collected gold-standard evidence sentences is 563, 207 positive and 356 negative sentences. We randomly selected the sentences from the PubMed abstracts that contain at least one event extracted using the Turku system and at least one gene symbol. As the numbers of negative sentences are much larger than those of positive sentences in the abstracts, we filtered out negative sentences to reduce the difference between numbers of positive and negative sentences. Note that the numbers of positive and negative sentences in the gold-standard data do not reflect those in the abstracts. We split the gold-standard data into feature selection and performance testing data sets. By examining the feature selection data set, we constructed features that might be contributing to separate positive sentences from negative sentences. By using the performance testing data, the proposed machine learning methods were trained and tested. Table 1 shows the number of evidence sentences for each event type, where gene expression events include positive and negative regulation events.

**Table 1.** Gold-standard data

Events	Binding	Gene expression	Local	Phosphorylation	Protein catabolism	Transcription
Positive	11 (18)	20 (52)	9 (19)	19 (18)	6 (8)	5 (22)
Negative	26 (29)	20 (46)	23 (38)	24 (38)	24 (17)	45 (26)
Total	37 (47)	40 (98)	32 (57)	43 (56)	30 (25)	50 (48)

Positive and negative gold-standard evidence sentences for binding, gene expression, localization, phosphorylation, protein catabolism and transcription are collected. For each event type, the numbers of feature selection (performance testing) sentences are shown.

### A method for scoring evidence sentences

Our approach is to assign higher scores to positive sentences and lower scores to negative sentences, so that positive sentences are displayed before negative sentences in the search results and furthermore so that more likely evidence sentences are displayed before less likely ones. To distinguish positive and negative sentences, 10 linguistically motivated features were constructed using the feature selection sentences. These features were obtained from ABNER and Turku systems, and dependency parse trees generated by Stanford parser (27), and based on hand-crafted cancer-related terms, and terms related to negative sentences.

- **Event and edge scores** These two features are from the Turku event extraction system. The Turku system detects and scores an event keyword and the relation between a gene and the keyword using an SVM, termed as an event SVM score and an edge SVM score, respectively. These values were normalized using the maximum SVM score of sentences in search results.
- **Distance among terms** An evidence sentence can contain four terms: a gene name, an event keyword, a regulation term that represents the relationship between the gene and the event keyword and a cancer term. Three distances among the four terms are calculated based on the dependency tree of the sentence as follows: gene–event distance, event–regulation distance and event–cancer distance. If any of the terms are not contained in evidence sentences, a penalty value is assigned instead of distance. In Sentence 1, a gene name ‘SOX9’, an event keyword ‘down-regulation’ and a cancer term ‘prostate cancer’ are detected, and a penalty value is assigned instead of event–regulation distance due to the absence of a regulation term.
- **Cancer keyword count** This feature counts the number of cancer terms or cancer-related keywords in the sentence. For this purpose, hyponyms and hypernyms of cancer were collected from WordNet (30). For example, Sentence 1 has value of one for this feature. Total 12 terms including cancer, tumor and carcinoma were used to construct this feature. This feature is useful when a query cancer name is not included in the sentence.
- **Hallmark keyword count** Keywords of known characteristics of cancer during its initiation, development and progress were used as a feature. The following six terms are cancer hallmark keywords obtained from a textbook (31): apoptosis, angiogenesis, growth, invasion, metastasis and proliferation. For example, Sentence 1 contains two such terms, growth and proliferation.
- **Event depth** This feature indicates the depth of the event keyword from the root in the dependency tree. It is useful to understand if the event is the main theme of the sentence. In Sentence 1, this feature has a value of two because the event term is located at the two-distance away from the root.
- **Negative score** This feature consists of phrases to detect negative sentences, including certain to-infinitive phrases (e.g. ‘to determine’, ‘to find’, ‘to assess’), the troponyms of ‘study’ from WordNet and such negation words as ‘not’ and ‘never’. Those to-infinitive phrases and the study words are often used to explain the purpose or methods of research, rather than to describe experiment results. The negation words often negate the relationship between gene and disease, thus classifying sentences into negative evidence sentences.
- **Agent** Our goal is to find the relationship between disease and gene, not between genes. Thus, if a sentence only contains events for the gene–gene relationship, it is likely to be classified as a negative evidence sentence. The genes that are expressed to have relations with a query gene in a candidate evidence sentence are called agents in this article. To check for the existence of an agent in a candidate sentence, we find an event, which is a parent of a query gene in the dependency tree of the sentence. If any gene other than the query gene is another child of the event, then it is regarded as an agent gene. In Sentence 1, when ‘AR’ is a query gene and ‘reduced’ is an event keyword, SOX9 is detected as the agent because SOX9 is located in the other branch of the event.

The lists of cancer-related keywords and terms related to negative score are provided in the Supplementary page.

A Bayesian classifier with these features was modeled to identify positive evidence sentences from negative sentences. By assigning the same prior to positive and negative evidence sentences, we calculate a likelihood ratio of features.
(1)
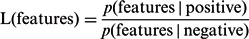

The naive Bayesian approach, which assumes conditional independence among features, works well in general despite its simplicity. After analyzing the feature selection data set, however, we empirically chose to add two types of dependencies, a dependency between cancer keyword and event–cancer distance and a dependency between agent and hallmark keyword, due to the following observations: The presence of a cancer keyword in a candidate evidence sentence plays an important role in approving the sentence when the event–cancer distance is large, which means that the cancer name does not co-occur with the event keyword in the candidate evidence sentence; and whereas the presence of an agent usually puts more weight on negative sentences, it does not do so when a hallmark appears in the candidate evidence sentence. The likelihood ratio can be rewritten as follows:
(2)
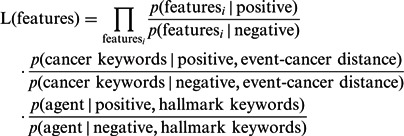

Candidate evidence sentences are ranked based on the likelihood of features. We expect that sentences with higher values are more likely to be positive evidence sentences. However, we do not have a threshold to filter out low-ranked candidates. Therefore, its results would include negative evidence sentences with less relevant events, expectedly at the bottom of the ranked list. In case of Localization, we applied an additional step to rank relevant events at higher position. According to BioNLP’09 and ’11 Shared Tasks, Localization represents a change of the location or the presence of a protein, so that the presence of a protein is often captured by the Turku event extraction system. However, we see that sub-cellular localization information is more important for the gene–disease association analysis, and thus, as a post-processing step, we placed sentences with sub-cellular location higher than other sentences by decreasing the relevance score of the evidence sentence without sub-cellular location information. This post-processing step was applied to develop and evaluate the DigSee system. In constructing DigSee system, a score from the Bayesian classifier was converted into a value in the range between 0 and 1 using a sigmoid function.

For comparison, a support vector machine model with bag of words was used. This model represents a text as a set of words, ignoring word order and frequency. We construct the bag of words model using gold-standard feature selection data. After excluding stop-words and words appearing once, 239 words were identified. A libsvm library was used for implementation of the SVM method.

### Accuracy of the proposed method for scoring evidence sentences

Performance of the proposed method was measured by 5-fold cross-validation using the performance testing data, in terms of both F-measure from precision and recall and area under the curve (AUC) score of true-positive and false-negative rate. We applied cut-off values to classify the top *k* ranked sentences, for all possible values of *k*, into positive sentences and the remaining sentences into negative sentences. For each cut-off value, the average F-measure of 5-fold cross-validation was calculated. Then, the cut-off value giving the highest average F-measure was selected for reporting the performance.

Table 2 shows accuracies of individual features. The event–cancer distance and cancer keywords count were the most useful features, having AUC values larger than 70%. Several features such as ‘Event depth’ did not show high performance on the testing data. Even though these poor-performing features may not be highly useful in general, we did not remove them because they did not decrease the performance of the system when they were integrated with other features. Also, when accuracies of ‘Cancer keywords count’ and ‘Agent’ were measured without conditional dependencies on ‘Event-cancer distance’ and ‘Hallmark keywords count’, respectively, AUC values were decreased to 68.8% and 53.4%, respectively. This confirms the importance of these two dependences. Note that although F-measures and AUC scores represent different aspects of the system performance, these two measurements produce similar relative orders of individual features; features with higher F-measure have higher AUC values, and vice versa, giving positive correlation of 92.8% between the values of two measurements. When all features were combined, the accuracy achieved an AUC value of 80.5% and an F-measure of 72.7% (precision 62.6% and recall 86.9%).

**Table 2.** Accuracies of individual features using performance testing data

Features	F-measure	AUC
Normalized event SVM score	62.7	57.8
Normalized edge SVM score	60.3	42.5
Gene–event distance	62.1	52.0
Event–regulation distance	64.5	59.7
Event–cancer distance	71.5	74.1
Cancer keywords count (depending on event–cancer distance)	72.5	72.5
Hallmark keywords count	64.4	58.8
Event depth	60.2	47.7
Negative score	68.7	59.3
Agent (depending on hallmark keywords count)	64.1	62.6
Total	72.7	80.5

To check whether the training data used are large enough to build robust models for the identified features, we tested the Bayesian classifier using different numbers of training data, and results are shown in the Supplementary page of the DigSee Web site. We found that the relative order of feature performance is not related to the size of training data, and that the performances of the features do not change so much, although slightly increasing with a larger number of data.

Table 3 shows the performance of the classifier models to classify positive and negative evidence sentences that are associated with each of the six biological event types. Binding shows the highest accuracy and Localization the lowest accuracy. Also, the Bayesian classifier using all the 10 features outperformed the SVM classifier using bag of words. A baseline method, called ‘random order’, was compared with our proposed method. In this method, sentences in the performance testing data were randomly ordered 100 times and the average performance was assessed. In the random order, precisions may be close to the ratio of positive evidence sentences and consistent for any cut-off values due to the random distribution of positive sentences, so that F-measure, the harmonic mean of precision and recall, often shows the highest value when recall is close to 100%. For instance, the random order method shows, for the event type of gene expression, 54% precision (similar to the ratio of positive sentences in Table 1), 99% recall (as explained previously) and 70% F-measure. The random order method may show a high F-measure for an event type, if the ratio of positive evidence sentences of the event type is high. Note that our proposed method outperforms the random order method for all the event types, as shown in Table 3.

**Table 3.** Accuracies of biological events

Biological events	Bayesian classifier				SVM classifier				Random order			
	P	R	F	AUC	P	R	F	AUC	P	R	F	AUC
Binding	80.0	91.2	83.6	87.1	45.7	88.3	60.0	62.6	42.2	94.1	57.8	50.7
Gene expression	66.7	96.2	78.7	79.3	65.7	88.7	75.5	73.7	54.4	98.6	70.0	50.7
Localization	66.7	53.3	59.0	72.5	56.0	75.0	63.9	71.5	37.5	90.7	52.1	50.5
Phosphorylation	75.0	85.0	79.3	93.7	90.0	51.7	65.3	75.2	37.2	90.0	51.4	50.9
Protein catabolism	100.0	70.0	80.0	96.7	40.0	80.0	52.0	61.7	42.6	87.4	55.4	52.0
Transcription	63.3	87.0	73.1	74.7	54.3	86.0	66.4	71.0	48.5	97.2	64.5	48.6
Total	62.6	86.9	72.7	80.5	53.7	81.0	64.5	71.2	43.7	93.0	59.4	49.8

In the table, a precision is shortened to ‘P’, a recall to ‘R’ and an F-measure to ‘F’.

We further evaluated the constructed DigSee system with a query of all genes and all event types for each of four cancer types (glioblastoma, breast cancer, pancreatic cancer and prostate cancer). We examined the top 10 sentences retrieved by each of the four queries and found that 6, 5, 5 and 8 sentences among them describe valid relations between genes and diseases, respectively, altogether showing the average precision of 60.0%, which is similar to the precision in Table 3. These top 10 sentences from four cancer types and gold-standard sentences are provided in the Supplementary page.

## DISCUSSION AND CONCLUSION

DigSee is a search engine to find explicit association between genes and cancer through biological events. Although several Web-based text-mining systems are available to extract implicit relationship between genes and cancer, most of them do not provide fine-grained information about the molecular context of gene–disease association. DigSee is a unique system in this aspect, and its accuracy was validated using manually curated evidence sentences.

Although DigSee may provide a new insight in searching for a gene–disease relationship, its performance can be improved further. When we examined the negative sentences among the query results for the four cancer types in the Supplementary page, we found that they often include descriptions about experimental procedures and association of a gene with another gene/phenotype/drug, but not with the disease in question. Also, incorrectly identified biological events were included in the negative sentences. Therefore, an important future work would be to further improve the performance of the ranking method. We will continue to work on filtering out irrelevant events. Also, we may incorporate inter-sentence dependencies such that similar sentences are assigned synergistically higher scores and contradicting sentences are assigned lower scores.

As the coverage of DigSee depends on biological event types supported by the system, we will incorporate more biological events such as methylation, alternative transcripts and single nucleotide polymorphisms into the system in the future work. Although LSAT (32) provides a Web service to identify sentences containing alternative transcripts for a given gene, the relation of alternative transcripts in disease was not provided. GeneView (9) allows a combined query of genes, single nucleotide polymorphisms and disease. However, it was designed for general purposes rather than for explicit triple relationships.

The graphs of gene co-occurrences from our current system can be further enhanced, borrowing ideas from other tools such as STRING (33), PESCADOR (34) and iHOP (35) that also provide services to explore relationships between genes. For example, PESCADOR builds a co-occurrence network and filters the network based on a given biological concept, whereas it is not specialized to the biological event level. We can narrow down the DigSee graphs to show the relationships between genes sharing the same molecular events.

In the future work, we will incorporate more disease types other than cancers into the system. Among features for the Bayesian classifier, ‘Cancer keywords count’ and ‘Hallmark keywords count’ are specific to cancer and are useful in classification, as shown in Table 2. To maintain the current performance of DigSee, it is necessary to provide these two features for other disease types. Whereas we can similarly use WordNet (30) for the other diseases in the case of ‘Cancer keywords count’, the ‘Hallmark keywords count’ were manually collected for the cancers. We will develop an automatic method to find hallmark keywords for the other diseases, for example, by identifying relatively frequent keywords in the collection of articles related to a given disease.

## FUNDING

Funding for open access charge: National Research Foundation of Korea (NRF); Korean government (MEST) [No. 2011-0029447].

*Conflict of interest statement.* None declared.

## References

[1] Safran M, Dalah I, Alexander J, Rosen N, InyStein T, Shmoish M, Nativ N, Bahir I, Doniger T, Krug H (2010). GeneCards Version 3: the human gene integrator. Database.

[2] Stenson P, Mort M, Ball E, Howells K, Phillips A, Thomas N, Cooper D (2009). The human gene mutation database: 2008 update. Genome Med..

[3] Essack M, Radovanovic A, Schaefer U, Schmeier S, Seshadri S, Christoffels A, Kaur M, Bajic V (2009). DDEC: Dragon database of genes implicated in esophageal cancer. BMC Cancer.

[4] Maqungo M, Kaur M, Kwofie S, Radovanovic A, Schaefer U, Schmeier S, Oppon E, Christoffels A, Bajic V (2011). DDPC: dragon database of genes associated with prostate cancer. Nucleic Acids Res..

[5] Cheng D, Knox C, Young N, Stothard P, Damaraju S, Wishart D (2008). PolySearch: a web-based text mining system for extracting relationships between human diseases, genes, mutations, drugs and metabolites. Nucleic Acids Res..

[6] Fontaine J, Priller F, Barbosa-Silva A, Andrade-Navarro M (2011). Génie: literature-based gene prioritization at multi genomic scale. Nucleic Acids Res..

[7] Tsuruoka Y, Tsujii J, Ananiadou S (2008). FACTA: a text search engine for finding associated biomedical concepts. Bioinformatics.

[8] Perez-Iratxeta C, Bork P, Andrade-Navarro M (2007). Update of the G2D tool for prioritization of gene candidates to inherited diseases. Nucleic Acids Res..

[9] Thomas P, Starlinger J, Vowinkel A, Arzt S, Leser U (2012). GeneView: a comprehensive semantic search engine for PubMed. Nucleic Acids Res..

[10] Knox C, Law V, Jewison T, Liu P, Ly S, Frolkis A, Pon A, Banco K, Mak C, Neveu V (2011). DrugBank 3.0: a comprehensive resource for ‘omics’ research on drugs. Nucleic Acids Res..

[11] Fang Y, Lai P, Dai H, Hsu W (2011). Meinfotext 2.0: gene methylation and cancer relation extraction from biomedical literature. BMC Bioinformatics.

[12] Ongenaert M, VanNeste L, DeMeyer T, Menschaert G, Bekaert S, VanCriekinge W (2008). PubMeth: a cancer methylation database combining text-mining and expert annotation. Nucleic Acids Res..

[14] Björne J, Heimonen J, Ginter F, Airola A, Pahikkala T, Salakoski T (2009). Extracting complex biological events with rich graph-based feature sets.

[15] Jia Z, Wang Y, Sawyers A, Yao H, Rahmatpanah F, Xia X, Xu Q, Pio R, Turan T, Koziol J (2011). Diagnosis of prostate cancer using differentially expressed genes in stroma. Cancer Res..

[16] Kim J, Baek S, BottoneFG J, Sali T, Eling T (2005). Overexpression of 15-lipoxygenase-1 induces growth arrest through phosphorylation of p53 in human colorectal cancer cells. Mol. Cancer Res..

[17] Vieyra D, Senger D, Toyama T, Muzik H, Brasher P, Johnston R, Riabowol K, Forsyth P (2003). Altered subcellular localization and low frequency of mutations of ING1 in human brain tumors. Clin. Cancer Res..

[18] Tejada S, Lobo M, García-Villanueva M, Sacristán S, Pérez-Morgado M, Salinas M, Martín M (2009). Eukaryotic initiation factors (eIF) 2alpha and 4E expression, localization, and phosphorylation in brain tumors. J. Histochem. Cytochem..

[19] Settles B (2005). Abner: an open source tool for automatically tagging genes, proteins and other entity names in text. Bioinformatics.

[20] Yeh A, Morgan A, Colosimo M, Hirschman L (2005). BioCreAtIvE task 1A: gene mention finding evaluation. BMC Bioinformatics.

[21] Leaman R, Gonzalez G (2008). Banner: an executable survey of advances in biomedical named entity recognition. Pac. Symp. Biocomput..

[22] Neves M, Carazo J, Pascual-Montano A (2010). Moara: a java library for extracting and normalizing gene and protein mentions. BMC Bioinformatics.

[23] Morgan A, Lu Z, Wang X, Cohen A, Fluck J, Ruch P, Divoli A, Fundel K, Leaman R, Hakenberg J (2008). Overview of BioCreative II gene normalization. Genome Biol..

[24] Magrane M, Consortium U (2011). UniProt Knowledgebase: a hub of integrated protein data. Database.

[25] Gray K, Daugherty L, Gordon S, Seal R, Wright M, Bruford E (2013). Genenames.org: the HGNC resources in 2013. Nucleic Acids Res..

[26] Hakenberg J, Plake C, Leaman R, Schroeder M, Gonzalez G (2008). Inter-species normalization of gene mentions with GNAT. Bioinformatics.

[27] DeMarneffe M, MacCartney B, Manning C (2006). Generating typed dependency parses from phrase structure parses.

[28] Wang H, McKnight N, Zhang T, Lu M, Balk S, Yuan X (2007). Sox9 is expressed in normal prostate basal cells and regulates androgen receptor expression in prostate cancer cells. Cancer Res..

[29] Wang L, Wu G, Yu L, Yuan J, Fang F, Zhai Z, Wang F, Wang H (2006). Inhibition of cd147 expression reduces tumor cell invasion in human prostate cancer cell line via RNA interference. Cancer Biol. Ther..

[30] Miller G, Beckwith R, Fellbaum C, Gross D, Miller K (1990). Introduction to wordnet: an on-line lexical database. Int. J. Lexicography.

[31] Pecorino L (2012). Molecular Biology of Cancer: Mechanisms, Targets, and Therapeutics.

[32] Shah P, Bork P (2006). LSAT: learning about alternative transcripts in MEDLINE. Bioinformatics.

[33] Jensen L, Kuhn M, Stark M, Chaffron S, Creevey C, Muller J, Doerks T, Julien P, Roth A, Simonovic M (2009). STRING 8–a global view on proteins and their functional interactions in 630 organisms. Nucleic Acids Res..

[34] Barbosa-Silva A, Fontaine J, Donnard E, Stussi F, Ortega J, Andrade-Navarro M (2011). PESCADOR, a web-based tool to assist text-mining of biointeractions extracted from PubMed queries. BMC Bioinformatics.

[35] Hoffmann R, Valencia A (2004). A gene network for navigating the literature. Nat. Genet..

